# Case Report: Debridement with vacuum sealing drainage for conservative management of post-cranioplasty surgical site infection

**DOI:** 10.3389/fsurg.2026.1847180

**Published:** 2026-07-28

**Authors:** Tong Ma, Chen Yang, Zhi Zheng, Xufeng Meng, Yunfei Hao

**Affiliations:** 1 First Clinical Medicine College, Gansu University of Traditional Chinese Medicine, Lanzhou, China; 2 Key Laboratory of Cerebrovascular Diseases of Gansu Province, Cerebrovascular Disease Center, Gansu Provincial People’s Hospital, Lanzhou, China

**Keywords:** cranioplasty, debridement, implant retention, infection, methicillin-resistant Staphylococcus aureus, polyetheretherketone, vacuum sealing drainage

## Abstract

Post-cranioplasty infection represents a challenging complication in neurosurgical practice. Conventional therapeutic strategies typically necessitate implant removal or decompressive craniectomy, often resulting in secondary trauma and increased healthcare burden. We report a 55-year-old male patient who underwent decompressive craniectomy following traumatic brain injury and subsequently received cranioplasty with polyetheretherketone (PEEK) material. Four months postoperatively, he presented with wound dehiscence and purulent discharge, and was diagnosed with combined intracranial and scalp infection caused by methicillin-resistant Staphylococcus aureus (MRSA). Following rigorous patient selection, two sessions of precise debridement were performed, combined with vacuum sealing drainage (VSD) therapy and individualized antimicrobial regimen based on susceptibility testing, with the PEEK implant preserved throughout. The infection was rapidly controlled, the VSD device was removed on postoperative day 6 with favorable wound healing, and no recurrence was observed at 3-month follow-up. Neurological function was fully restored with a modified Rankin Scale score of 0. This case demonstrates that for patients with post-cranioplasty infection involving PEEK material, a comprehensive approach incorporating debridement, VSD drainage, and targeted antimicrobial therapy—following strict evaluation—can achieve optimal outcomes with implant retention, offering a conservative treatment paradigm for such complex cases.

## 1 Introduction

Cranioplasty represents a commonly performed neurosurgical procedure for patients undergoing decompressive craniectomy following trauma, characterized by favorable safety profiles. This intervention not only restores cranial morphology and protective function, improves cosmetic appearance and psychological status, but also facilitates the normalization of postoperative cerebrospinal fluid dynamics (1, 2). By reconstructing cranial architecture, restoring cerebrospinal fluid circulation, and enhancing cerebral perfusion, cranioplasty effectively alleviates cognitive and neurological deficits in affected patients (3). With advances in biomaterial development, polyetheretherketone (PEEK) has emerged as an ideal material for complex cranial defect reconstruction owing to its excellent mechanical strength and biocompatibility, providing stable protection for the defective region (4). Postoperative infection is a severe problem affecting prognosis, with high mortality and disability rates (5). According to domestic and international data, the incidence of intracranial infection following craniotomy is approximately 2.6% in China and 0.5% in the United States (6, 7). Traditional management predominantly advocates removal of the infected bone flap with delayed secondary repair, primarily because long-term cranial defects are prone to cause secondary brain injury and cosmetic deformity (8). Intracranial infection is one of the most dangerous complications following craniotomy, which can rapidly lead to meningitis, ventriculitis, brain abscess, and other severe pathological conditions (9, 10). Despite continuous improvements in surgical techniques and aseptic protocols, postoperative intracranial infection remains difficult to completely avoid (11). Major risk factors include prolonged operative time, indwelling drainage catheters, artificial material implantation, cerebrospinal fluid leakage, as well as diabetes mellitus and hypertension (12). Following infection, bacteria readily adhere to the surface of reconstructive materials, forming biofilms that render antimicrobial therapy and conventional debridement inadequate for complete pathogen eradication. According to traditional protocols, early removal of the reconstructive material is indicated upon infection, with decompressive craniectomy performed when necessary for infection control (13). However, PEEK material extraction subjects patients to additional surgical trauma and economic burden, while potentially increasing risks of complications such as intracranial hemorrhage and cerebral tissue injury. Furthermore, decompressive craniectomy disrupts the physiological cranial architecture, predisposing patients to long-term complications including cerebrospinal fluid circulation disturbances and cerebral tissue displacement (3). Currently, there have been no reports of successful treatment of postoperative infection following PEEK cranioplasty with implant retention,and the feasibility, safety, and applicable indications of such strategies have not reached consensus. The present report discusses comprehensive treatment strategies for implant retention based on our successful management of this case, offering clinical reference for similar scenarios.

## 2 Case presentation

**Figure 1:**
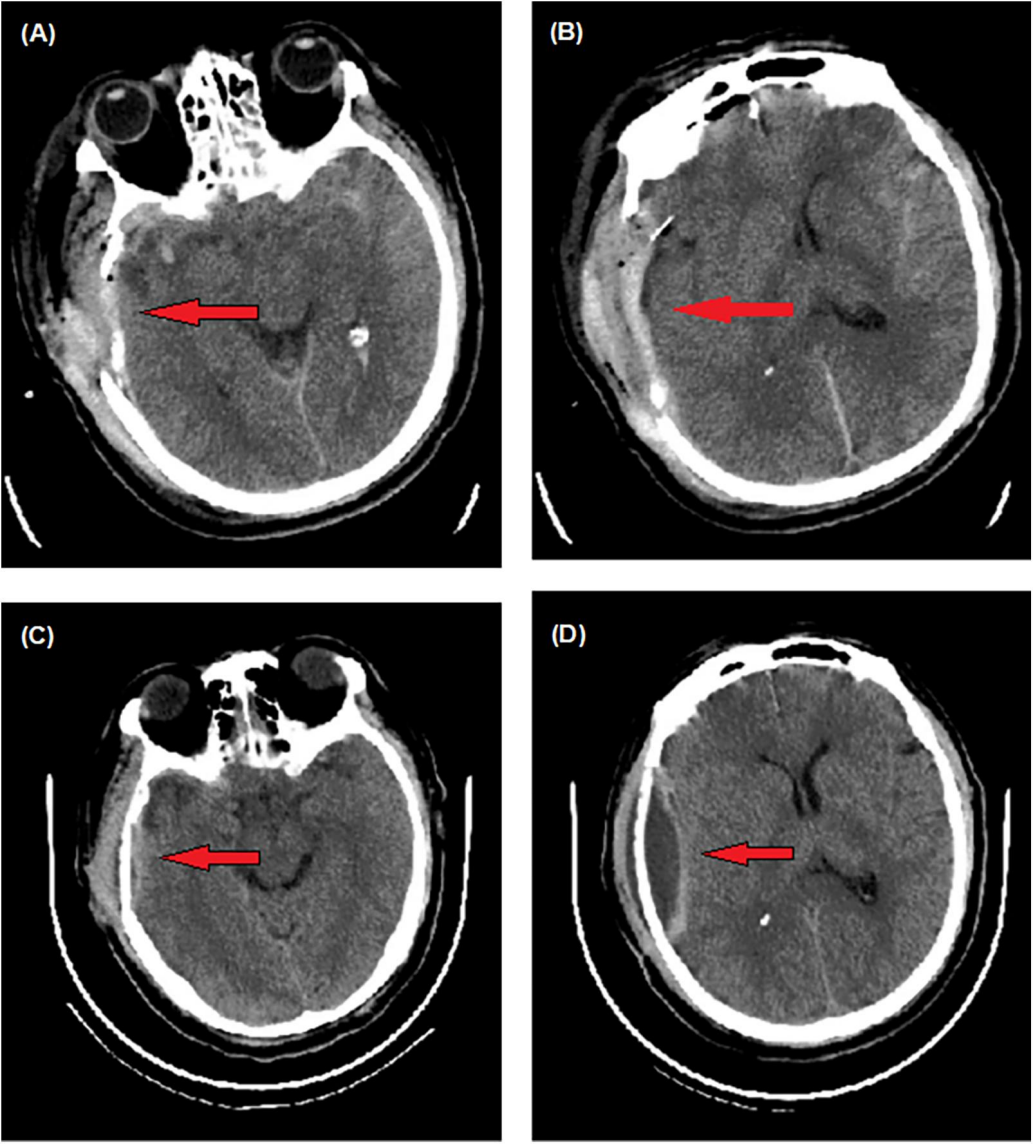
Imaging findings before and after cranioplasty. **(A,B)** Axial computed tomography (CT) images acquired on March 4, 2025: bilateral intracranial heterogeneous hyperdense and hypodense lesions with compression and narrowing of the right lateral ventricle; hyperdense casting in the left cerebral sulci and cisterns; and increased density of the falx cerebri and tentorium cerebelli. **(C,D)** Axial CT images acquired on June 23, 2025: heterogeneous fluid-density collection with mixed hyperdense and hypodense components in the surgical region, minimal intracranial and extracranial pneumocephalus; hypodense lesion in the right frontal lobe; and subcutaneous soft tissue swelling in the surgical region.

A 55-year-old male patient underwent decompressive craniectomy for traumatic subdural hemorrhage on March 4, 2025 (Figures 1A,B), with uneventful postoperative recovery. On May 12, 2025, he received right frontoparietal cranioplasty with polyetheretherketone (PEEK) material (Figures 1C,D). The procedure was uneventful, and the patient was discharged following primary wound healing. On July 4, 2025, he was admitted for wound dehiscence with purulent discharge one day after cranioplasty. On July 3, 2025, the patient developed spontaneous skin breakdown at the previous surgical site with yellow purulent exudate, without chills, fever, dizziness, headache, nausea, vomiting, motor deficits, or altered consciousness. He had no history of diabetes, hypertension, immunodeficiency, or other comorbidities. He had undergone open reduction and internal fixation for right forearm fracture three years previously, with the plate retained.

**Figure 2:**
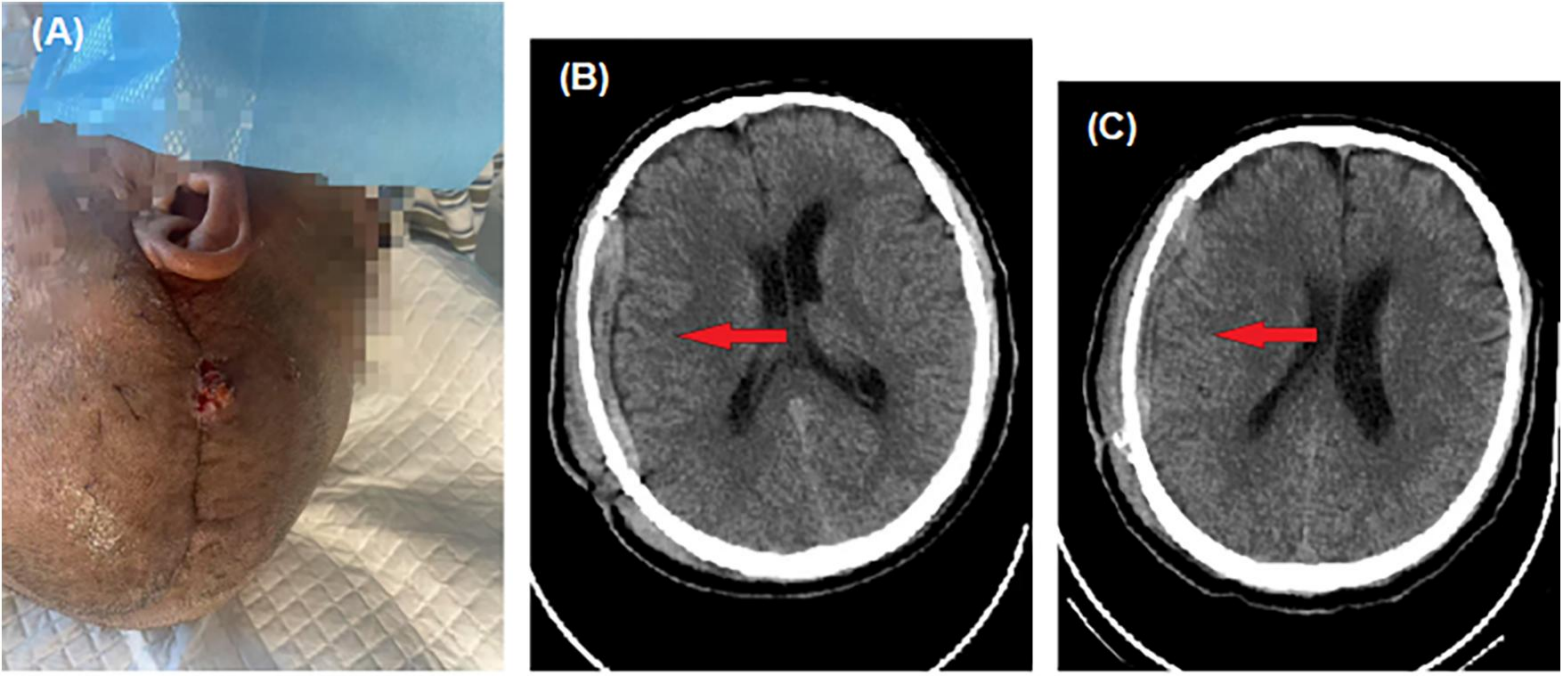
Clinical and imaging findings at infection presentation. **(A)** Clinical photograph demonstrating wound dehiscence with purulent discharge. **(B)** Axial computed tomography (CT) image acquired on July 4, 2025, prior to initial debridement: subcutaneous soft tissue swelling and inflammatory exudate in the surgical region (red arrow). **(C)** Axial CT image acquired on July 7, 2025, following initial debridement: significant resolution of soft tissue swelling and near-complete absorption of inflammatory exudate.

Infection Manifestations: On July 3, 2025, skin breakdown was observed at the right frontoparietal cranioplasty incision (Figure 2A) with yellow purulent discharge. On July 4, 2025, laboratory investigations revealed elevated neutrophil proportion and count, wound culture yielded methicillin-resistant Staphylococcus aureus (MRSA), non-contrast head computed tomography (CT) demonstrated post-cranioplasty changes in the right frontotemporoparietal region, with localized subcutaneous soft tissue swelling, minimal subdural fluid and hemorrhage collection, stable PEEK material position, no cerebral edema or mass effect, and no definitive brain abscess or diffuse meningitis (Figure 2B).

**Figure 3:**
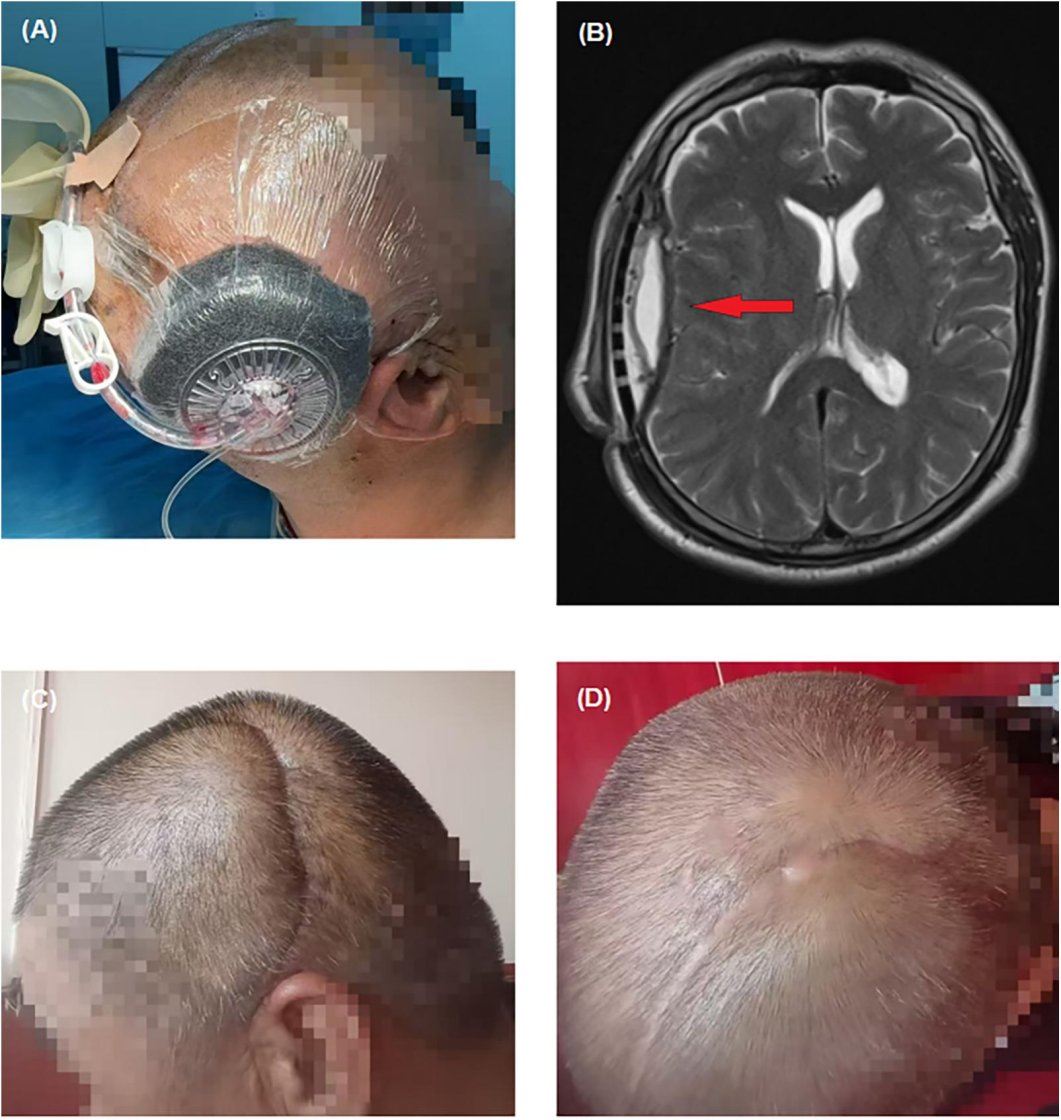
Vacuum sealing drainage application and follow-up outcomes. **(A)** Clinical photograph demonstrating vacuum sealing drainage (VSD) device placement. **(B)** Magnetic resonance imaging (MRI) showing resolution of scalp soft tissue swelling and inflammatory hyperintensity in the previously infected region, with uniform signal intensity across all layers and controlled acute inflammation; small epidural fluid collection in the right frontotemporal region, normal signal in subgaleal and other spaces without fluid or pus accumulation, suggesting cleared infection. **(C,D)** Three-month follow-up clinical photographs in sagittal and axial views demonstrating scalp recovery: no significant erythema, exudate, or dehiscence, with no evident infectious inflammation at the incision site.

Treatment Course: On July 4, 2025, the first debridement of the infected wound was performed under general anesthesia.Upon reopening the previous incision, abundant yellow purulent fluid was encountered. Hyperplastic granulation and necrotic tissues were debrided, with specimens obtained for microbiological examination. The surgical field was irrigated repeatedly with povidone-iodine saline and hydrogen peroxide, followed by local vancomycin solution irrigation. A drainage catheter was placed and the wound was closed in layers. Postoperative antimicrobial therapy comprised vancomycin combined with linezolid according to susceptibility results. Follow-up head CT demonstrated resolution of swelling, normalization of soft tissue and fat planes, significant absorption of inflammatory exudate, normal intracranial architecture, and no secondary infection (Figure 2C). On July 16, 2025, local erythema, swelling, and purulent exudate persisted at the incision site, indicating suboptimal infection control, indicating suboptimal infection control. Consequently, a second craniectomy wound debridement combined with vacuum sealing drainage (VSD) was performed under local anesthesia. The previous incision was reopened at the suppurative focus, with thorough debridement of residual infected tissue and purulent material. Following repeated irrigation of the abscess cavity, a VSD device was placed in the subcutaneous plane (Figure 3A). Continuous negative pressure drainage was maintained postoperatively, with continued vancomycin administration and regular monitoring of inflammatory markers including complete blood count and C-reactive protein, together with observation of granulation tissue growth.

Outcome: On July 17, 2025, wound exudate markedly decreased, body temperature normalized, and inflammatory markers gradually declined. Subsequent examination demonstrated normalization of inflammatory indices with fresh, well-granulating wound tissue. The VSD device was removed on postoperative day 6, with no erythema or exudate observed; the wound was covered with sterile gauze. On July 28, 2025, cranial MRI revealed that the infection was basically controlled and the scalp soft tissue swelling had significantly subsided (Figure 3B). On July 31, 2025, the patient was discharged with wound healing. Neurological examination at discharge revealed no abnormalities. Oral linezolid tablets were continued for one month post-discharge. At three-month follow-up (Figures 3C,D), the patient remained free of infection recurrence. Head CT demonstrated stable PEEK material position, well-defined scalp soft tissue layers, no cerebral edema, and restored activities of daily living with a modified Rankin Scale score of 0. In early June 2026, the patient was re-examined at the outpatient clinic of our hospital, and no obvious abnormality was found (Table 1).

**Table 1:** Timeline of clinical management.

Time Point	Clinical Event
March 4, 2025	Decompressive craniectomy for traumatic subdural hemorrhage
May 12, 2025	Right frontoparietal cranioplasty with polyetheretherketone (PEEK) following clinical stabilization
July 3, 2025	Wound dehiscence with purulent discharge, indicating infection
July 4, 2025	Hospital admission; emergency initial thorough debridement with local antibiotic irrigation
July 15, 2025	Persistent purulent exudate; secondary infectious focus debridement combined with vacuum sealing drainage (VSD)
July 21, 2025	Infection controlled; VSD device removed
July 28, 2025	Satisfactory wound healing; discharge with continued oral antimicrobial therapy
October 2025	Outpatient follow-up: no infection recurrence, complete neurological recovery

## 3 Discussion

The fundamental objectives in managing post-cranioplasty infection encompass complete eradication of the infectious source, control of inflammation, protection of cerebral tissue, and preservation of cranial structural integrity whenever feasible. This case challenges conventional therapeutic paradigms by achieving successful infection control with retained PEEK material. The innovative aspects and rationale are elaborated as follows. Traditional clinical perspectives generally maintain that once intracranial infection occurs, cranioplasty materials readily serve as substrates for bacterial biofilm colonization, particularly with resistant pathogens such as MRSA, necessitating material removal for definitive infection control. However, this patient presented multiple favorable conditions supporting material retention: PEEK demonstrates superior clinical outcomes and complication profiles compared with autologous bone flaps (4). With only two months since PEEK implantation, neither imaging studies nor intraoperative inspection revealed material surface corrosion or loosening, excluding the implant itself as the primary infectious focus. Furthermore, inherent material characteristics provided crucial support for retention therapy: PEEK exhibits excellent tolerance to gamma irradiation and electron beams, with favorable compatibility for ultrasonography and MRI, ensuring strong radiological adaptability (14). Compared with conventional titanium mesh, PEEK offers superior biocompatibility and smoother surface topography, rendering bacterial adherence and biofilm formation less likely, while avoiding metal ion release that might stimulate local tissue inflammation, thereby reducing persistent inflammatory triggers at the material level and facilitating infection control (4).

Based on routine bacterial smear, bacterial and fungal cultures, and qualitative antimicrobial susceptibility testing. MRSA was isolated from the secretion culture; microscopy revealed scant leukocytes and Gram-positive cocci, with fungal elements absent. The isolate demonstrated resistance to penicillin and oxacillin, while remaining susceptible to vancomycin and linezolid. *β*-Lactam antibiotics are not indicated. Regarding antimicrobial selection, methicillin-resistant Staphylococcus aureus (MRSA), first identified in 1961, has emerged as a predominant pathogen in healthcare settings. This organism causes diverse infections including wound infections, osteomyelitis, and pneumonia (15). In most circumstances, vancomycin remains the gold standard for MRSA infection treatment (16). However, given the limited penetration of glycopeptides into the central nervous system, linezolid has been repeatedly recommended for MRSA central nervous system infections due to its excellent penetration into brain tissue and cerebrospinal fluid (17, 18). Additionally, material retention avoids additional surgical trauma and economic burden associated with re-craniotomy and material customization (3), while reducing risks of secondary complications such as intracranial hemorrhage and cerebral tissue injury, aligning more closely with minimally invasive therapeutic principles. In clinical practice, decompressive craniectomy is frequently employed for intracranial infection complicated by elevated intracranial pressure and significant cerebral edema, aiming to reduce intracranial pressure and improve cerebral perfusion (19). The decision to forgo decompressive craniectomy and retain PEEK material in this case was well justified: the patient demonstrated stable intracranial pressure and cerebral status, with head CT showing no significant cerebral edema, and clinically exhibited no manifestations of elevated intracranial pressure such as headache, vomiting, or altered consciousness. Although the infection involved intracranial structures, it did not produce cranial pressure or structural imbalance, thereby obviating the need for decompressive craniectomy. Conversely, vacuum sealing drainage (VSD) technology offered a valuable alternative. This system, comprising VSD dressing, semipermeable membrane, three-way tubing, and negative pressure apparatus, has been widely applied in complex wound management including trauma, burns, osteomyelitis, pressure ulcers, and diabetic foot (20). VSD Device Main Components: PVA/PU porous foam dressing, unidirectional semi-permeable sealing film, multi-side-hole drainage tubing, and electric/central negative pressure source. Principle of Negative Pressure Formation: The sealing film creates an independent chamber over the wound surface; the negative pressure source continuously evacuates air from the chamber, generating a vacuum gradient below atmospheric pressure. Mechanisms for Long-term Vacuum Maintenance: Four integrated mechanisms ensure stable effective negative pressure for 5–14 days: unidirectional gas-blocking sealing film, highly permeable non-collapsible foam, automatic pressure-stabilizing compensation by the pump, and anti-reflux tubing plus irrigation anti-clogging system. Through continuous negative pressure suction, necrotic tissue and secretions are removed, offering minimally invasive advantages for deep-seated lesions while maximally preserving viable tissue (20, 21). In this case, precise debridement eliminated the infectious focus and purulent material, combined with continuous VSD drainage of residual bacteria and exudate, effectively alleviating local tissue tension and controlling infection spread without further compromising cranial integrity. This approach simultaneously avoided long-term risks associated with decompressive craniectomy, including cerebrospinal fluid dynamics disturbances and cerebral tissue displacement, achieving therapeutic objectives while minimizing unnecessary surgical trauma.

The critical factors contributing to successful treatment in this case were the integrated strategy of precise debridement, VSD drainage, and individualized antimicrobial therapy: two sessions of thorough debridement eliminated the infectious focus and secretions, reducing biofilm residue; continuous VSD drainage maintained wound dryness, preventing recurrent contamination (20); and selection of linezolid based on susceptibility testing, with demonstrated blood-brain barrier penetration, combined with intraoperative vancomycin irrigation, achieved targeted anti-MRSA therapy (17), ultimately realizing infection cure with preserved reconstructive material. Under the precondition of retaining the repair material, the aforementioned combined regimen may also be applicable to materials other than PEEK. Regarding VSD, previous literature has reported that vacuum sealing drainage (VSD)-assisted therapy is safe and effective against surgical site infections after intracranial neurosurgery (22).

## 4 Conclusion

Based on the therapeutic course of this patient, we identified that for intracranial infection following PEEK cranioplasty, achieving cure without material removal or decompressive craniectomy necessitates strict adherence to selection criteria: localized infection without manifestations of severe intracranial infection such as diffuse meningitis or brain abscess; absence of significant cerebral edema and essentially normal intracranial pressure; implantation duration not exceeding three months with no material surface erosion or loosening, intact dura mater, and no direct communication between the material and infectious focus; pathogen susceptibility to antimicrobial agents with blood-brain barrier penetration and absence of multidrug resistance; and absence of underlying conditions such as diabetes mellitus or immunodeficiency with preserved systemic anti-infective capacity. Throughout the treatment period, dynamic monitoring of cerebrospinal fluid routine examination, biochemical parameters, and bacterial culture, combined with timely radiological assessment, is essential. Immediate modification of therapeutic strategy is indicated upon development of infection spread, elevated intracranial pressure, or material loosening.

## Data Availability

The original contributions presented in the study are included in the article/Supplementary Material, further inquiries can be directed to the corresponding author.
